# Supplementary material for: Pupil-linked arousal is driven by decision uncertainty and alters serial choice bias
Source: Nat Commun. 2017 Mar 3;8:14637. doi: 10.1038/ncomms14637 (PMC5337963; doi:10.1038/ncomms14637)
Supplement: Supplementary Information — Supplementary Figures [file ncomms14637-s1.pdf]

SUPPLEMENTARY FIGURES

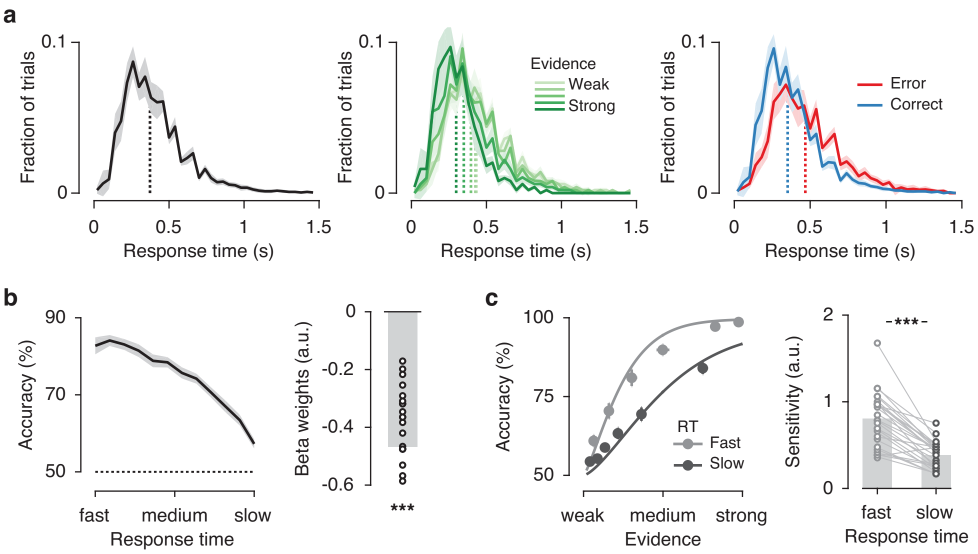

Supplementary Figure 1 | RTs scale with decision uncertainty. (**a**) RT distributions from stimulus offset, shown for all trials (left), split into five bins of evidence strength (middle), and separately for correct and error trials (right). For each observer, the number of trials was counted in each 40-ms wide bin from 0 to 1.5 seconds after stimulus offset, and normalised by the total number of trials. Shaded error bars indicate group median and inter-quartile range. Dotted line indicates group mean of individual RT medians. (**b**) RT predicted choice accuracy over a range from about 85% to about 60% correct, and not below chance level (50%). This relationship is consistent with decision uncertainty, but not error detection, which predicts accuracies of a range from 100% to 0% correct. Left: Accuracy for 12 bins of RT, shaded error bars indicate group mean ± s.e.m. Right: Individual logistic regression weights, using RT to predict single-trial accuracy. (**c**) Slow RTs reflected lower perceptual sensitivity. Left: Average cumulative Weibull psychometric function fits and data points (group mean ± s.e.m.), separately for the lowest and highest RT tertiles. Right: Individual perceptual sensitivity, separately for lowest and highest RT tertiles. In **b-c**, we z-scored and log-transformed RTs within each block and removed trial-to-trial variability shared with pupil responses via linear regression before computing statistics. *** $p < 0.001$, permutation test. (N=27, group mean ± s.e.m.)

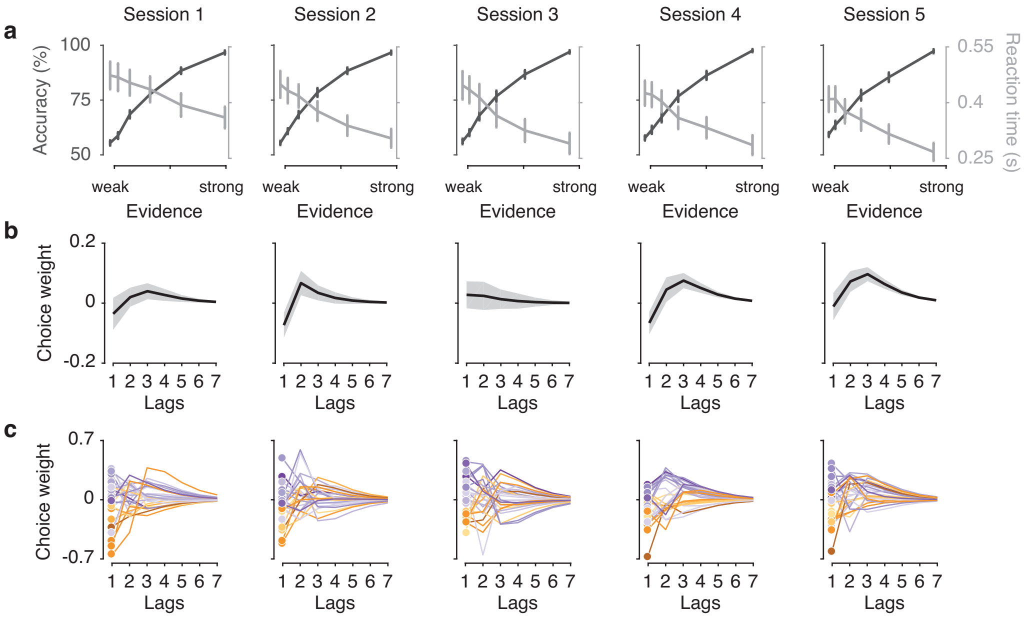

Supplementary Figure 2 | Behaviour over sessions. Data from each observer were collected over 5 main experimental sessions of 500 trials. Data from the practice session are not shown here. After discarding trials in which no response was recorded, each session contained an average of 498 trials (range 465-500). (**a**) Psychometric and chronometric functions, as in Figure 2d, separately for each session. (**b**) History kernels as in Figure 5c, separately for each session. (N=27, group mean ± s.e.m.) (**c**) Individual history kernels as in Figure 5c, separately for each session. Colours indicate the choice weight as derived from the model in Figure 5c,d, fit across all sessions combined.

To complement these visual representations of behaviour over sessions, we computed repetition probability for three bins of pupil responses (Figure 4a), separately in each of the five sessions. Using a repeated measures ANOVA, we found no main effect of session ($F_{(4,104)}$ = 1.591, p = 0.182, Bf_{10} = 0.078) nor an interaction between session and pupil bin ($F_{(8,208)}$ = 1.333, p = 0.229, Bf_{10} = 0.023) on repetition probability. This analysis indicates that history biases do not detectably change over the course of learning, adding further evidence to the idea that serial choice biases are stable, individual traits.

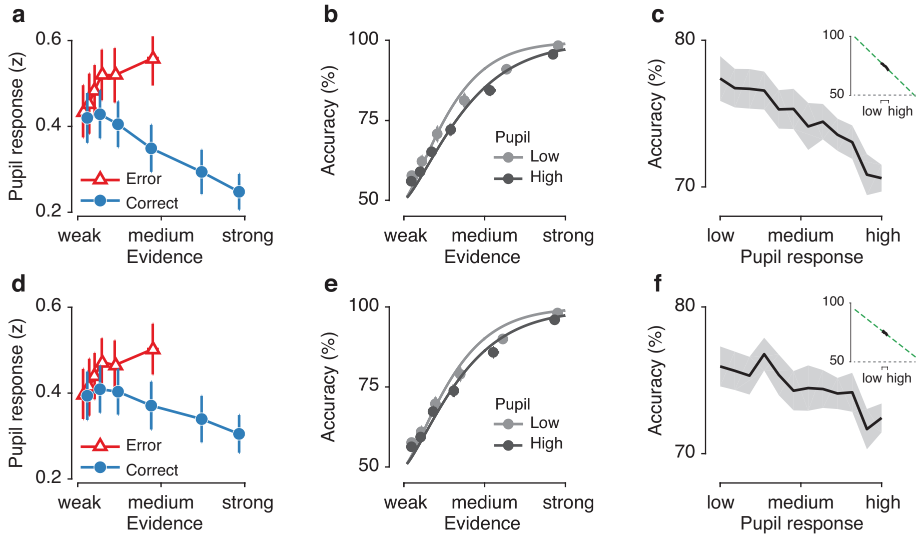

Supplementary Figure 3 | Pupil responses scale with decision uncertainty. Testing for all three signatures of decision uncertainty derived from the model in Figure 1. (**a**) Pupil responses scaled oppositely with evidence strength on correct and error trials. (**b**) High pupil responses reflected lower perceptual sensitivity. Average cumulative Weibull psychometric function fits (see Methods) and data points, separately for the lowest and highest tertiles of pupil responses. (**c**) Accuracy as a function of pupil responses (12 bins). Pupil responses predicted uncertainty over a range between 100% and 50% correct, but clearly not below 50% correct. This scaling is more consistent with decision uncertainty than with error awareness (which predicts accuracies down to 0%). Note that the analysis is limited by noise corrupting the single-trial pupil measurements. To address this issue, we fit a line to the data in **c**, extended its negative range to reach 100% accuracy, and then extended its positive range, with an equal distance. The result, shown in the inset, provided a rough estimate of the relationship expected, based on our result, if single-trial pupil-linked arousal could be measured without noise. Again, this analysis indicates that the scaling of pupil responses with accuracy is more consistent with decision uncertainty than with error awareness. (**d**-**e**) Same as **a**-**c**, after removing trial-by-trial fluctuations in log-transformed RT from the pupil signal using linear regression. The scaling of the pupil response with decision uncertainty was not inherited from the analogous scaling of RT. (N=27, group mean ± s.e.m.)

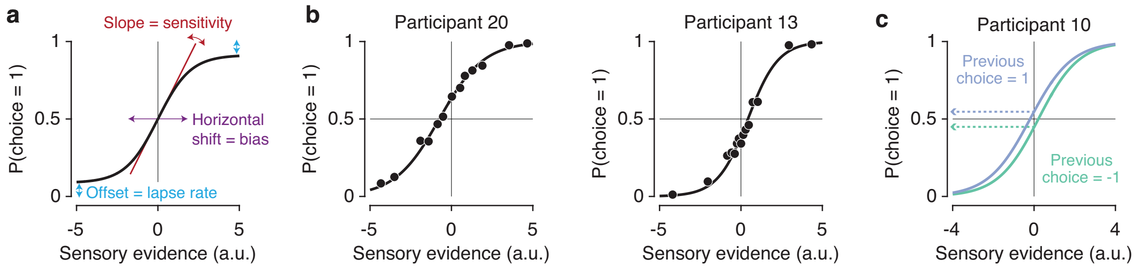

Supplementary Figure 4 │Quantifying choice bias using psychometric function fits. (**a**) A logistic psychometric function quantifies separate aspects of choice behaviour. The slope of the function indicates the observer's perceptual sensitivity. The intercept indicates a horizontal shift of the psychometric function, reflecting a bias towards a specific choice independent of the sensory evidence. The vertical offsets from the two asymptotes indicate the fraction of stimulus-independent errors ("lapses"). See also Methods. (**b**) Example psychometric functions with corresponding data points, for an example observer with a bias towards choice 1 (left) and an observer with a bias towards choice -1 (right). (**c**) History-dependent choice bias. Example observer with a tendency to repeat the previous choice.

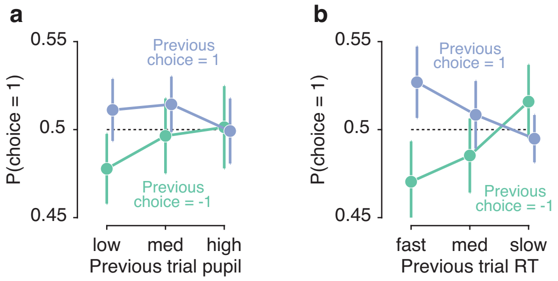

Supplementary Figure 5 │Pupil modulation of history-dependent choice bias. (**a**) Modulation of repetition probability by previous trial pupil response. P(choice = 1) was computed from the intercept of the logistic function (see Methods), for tertiles of previous trial pupil responses. (**b**) as in **a**, but for tertiles of previous trial RT. The two choice identities were collapsed to obtain the measure of repetition probability in Figure 4a and 4f. (N=27, group mean ± s.e.m.)

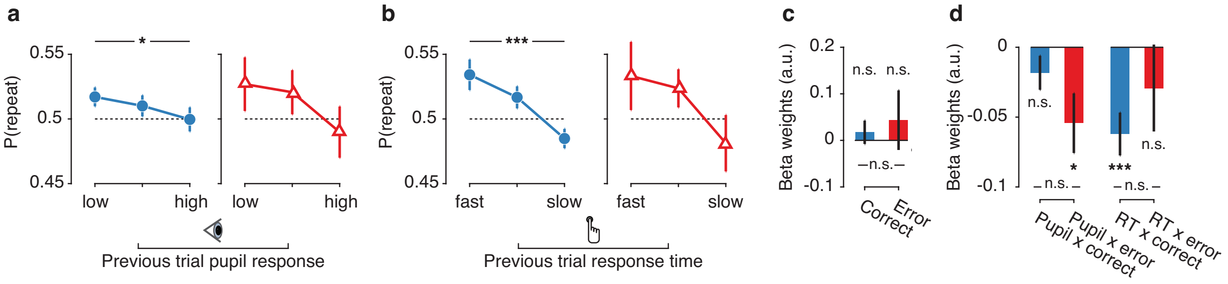

Supplementary Figure 6 │Pupil responses and RT modulate serial choice bias within categories of accuracy. (**a**) Repetition probability, for tertiles of previous trial pupil responses, separately for correct (left) and error (right) trials. (**b**) As in **a**, but for tertiles of previous trial RT. (**c**) Beta weights for repeating a previous correct vs. incorrect choice (see Methods). (**d**) Pupil- and RT modulation weights for repeating a previous correct vs. incorrect choice. Beta weights were obtained from the model show in Figure 5e-g, with pupil- and RT-linked modulatory terms included in the same regression model. Statistics indicate the main effect of a one-way ANOVA (**a**, **b**) or a permutation test (**c**, **d**). *** $p < 0.001$,* $p < 0.05$, n.s. $p > 0.05$. (N=27, group mean ± s.e.m.)

These results indicate that the modulatory effect of pupil responses (and RT) on serial choice biases was not purely driven by higher pupil responses on error trials. Instead, serial choice bias was modulated by trial-to-trial fluctuations in pupil-linked arousal within categories of trial outcomes.

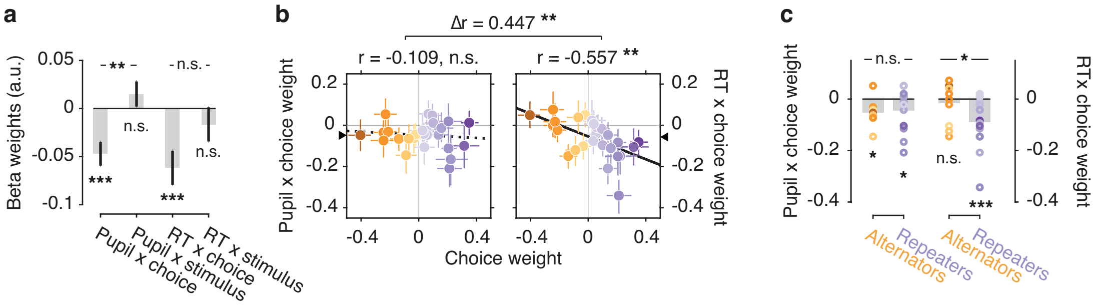

Supplementary Figure 7 │Modelling results do not depend on simultaneous fitting of both pupil and RT. Running two separate regression models, one including only pupil response and one only including RT as a modulatory variable, gives the same results as shown in Figure 5 (where the two were included in the regression model simultaneously). (**a-c**) as in Figure 5e-g, but with data obtained from two separate regression models.

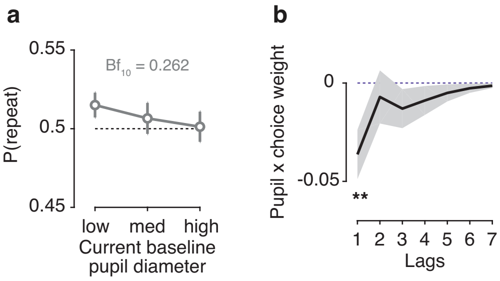

Supplementary Figure 8 │Predictive effect is specific for pupil response on the preceding trial. (**a**) Baseline pupil diameter on the current trial did not predict a modulation of serial choice bias. Repetition probability, for tertiles of current trial baseline pupil diameter (main effect of one-way repeated measured ANOVA, $F_{(2,52)}$ = 1.164, p = 0.320). (**b**) Pupil modulation of choice bias was only significant (** p < 0.01) across the group of observers at lag 1 (same data as Figure 5e), and did not reach significance beyond one trial in the past. This finding indicates that the modulation of choice biases by pupil responses was more short-lived than the overall serial choice biases shown in Figure 5c. (N=27, group mean ± s.e.m.)

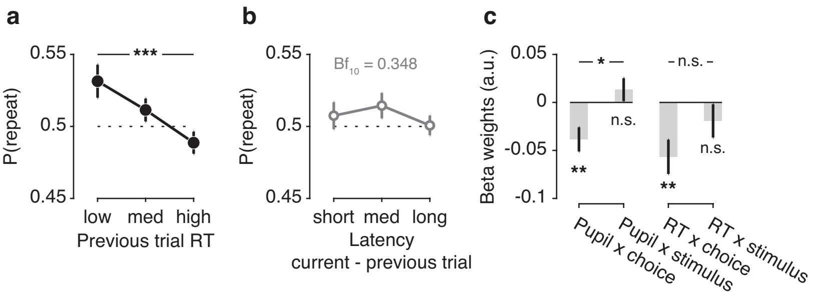

Supplementary Figure 9 │Serial choice biases are not explained by variations in interval timing. (**a**) To measure the passage of time between trials, we computed the latency between the onset of each test stimulus and the onset of the next trial's test stimulus. These latencies correlated with RTs (mean Spearman's rho 0.296, range 0.086 to 0.726). Removing these trial-by-trial latencies from RT (using linear regression) did not abolish the effect of RTs on serial choice bias (main effect of RT bin, $F_{(2,52)}$ = 10.846, p < 0.001, Bf_{10} = 225.756). (**b**) Latencies did not predict a modulation of serial choice bias (main effect of latency bin, $F_{(2,52)}$ = 1.541, p = 0.224, Bf_{10} = 0.349). These results suggest that the uncertainty component of RTs, rather than the passage of time between trials, modulated serial choice bias. (**c**) We tested whether the modulation of serial bias by pupil response could be explained by trial-to-trial variations in the jittered interval between s1 and s2, or between button press and feedback delivery. When these random variations were long, they could cause larger pupil responses, irrespective of the amplitude of the underlying neural input, by driving the peripheral pupil apparatus for a longer duration. We removed these trial-to-trial interval durations from pupil responses using linear regression, and reran the analysis shown in Figure 5e. Although pupil responses were weakly correlated to the interval between s1 and s2 (mean Spearman's rho -0.007, range -0.055 to 0.047, significant in 3 out of 27 observers) and the interval between button press and feedback (mean Spearman's rho 0.056, range -0.025 to 0.290, significant in 13 out of 27 observers), removing this variance from trial-by-trial pupil responses did not change the predictive effect of pupil responses on serial choice bias. Statistics indicate the main effect of a one-way ANOVA (**a**, **b**) and permutation test (**c**). *** p < 0.001, ** p < 0.01, * p < 0.05, n.s. p > 0.05. (N=27, group mean ± s.e.m.)

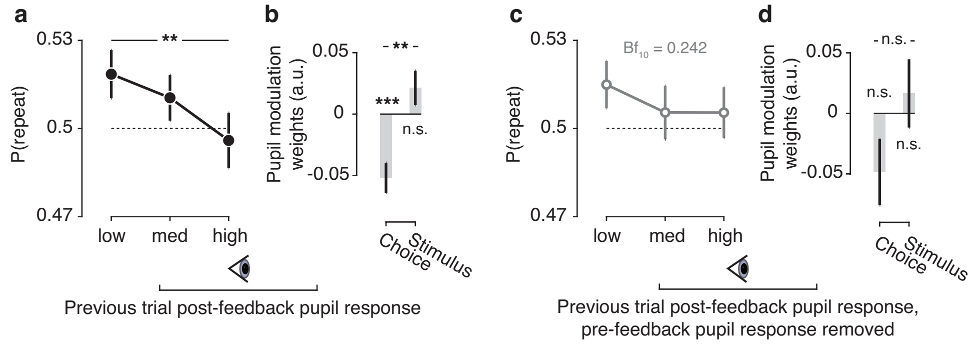

Supplementary Figure 10 | Serial choice biases are not explained by post-feedback pupil responses. To test whether serial choice biases were modulated by pupil responses to the feedback tone beyond pre-feedback uncertainty signalling, we computed post-feedback values as the mean pupil diameter 515-765 ms after feedback tone delivery. This window was defined as the peak of the grand average pupil response, and its length set equal to our pre-feedback window. (**a**) Serial choice bias for tertiles of previous trial post-feedback pupil responses (main effect of pupil bin, $F_{(2,52)} = 5.479$, $p = 0.007$, $Bf_{10} = 6.014$). (**b**) Beta weights for the interaction between previous trial post-feedback pupil response and choice or stimulus, as in Figure 5e. (**c**) We removed the effect of single-trial pre-feedback from the post-feedback signal using linear regression. The residual reflected the effect of feedback on uncertainty scaling in the pupil, after taking into account the scaling already present before the feedback tone. Serial choice bias, for tertiles of residual pupil responses (main effect of pupil bin, $F_{(2,52)} = 1.063$, $p = 0.353$) (**d**) Modulation weights for post-feedback pupil responses, with pre-feedback pupil responses added as a covariate in the same regression model. The information about serial biases was already contained in the pupil signal before feedback delivery. Statistics indicate the main effect of a one-way ANOVA (**a**, **c**) and permutation test (**b, d**). *** $p < 0.001$, ** $p < 0.01$, n.s. $p > 0.05$. (N=27, group mean ± s.e.m.)

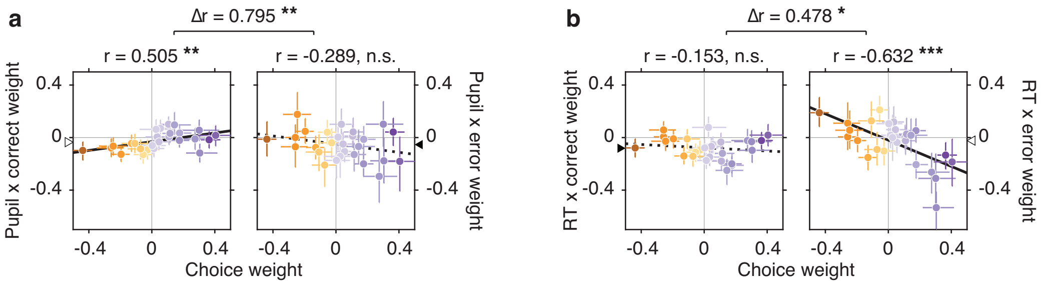

Supplementary Figure 11 │Differential gating of individual choice modulation by trial outcome. Pupil-and RT-linked modulations of serial choice bias were differentially gated by trial outcome. We computed correct and error modulation weights from choice and stimulus modulation weights (see Methods). (**a**) Correlation between choice weights and pupil modulation weights, separately for correct and incorrect choices. (**b**) Correlation between choice weights and RT modulation weights, separately for correct and incorrect choices. Colours indicate the choice weight as derived from the basic model in Figure 5c. Error bars indicate a 68% confidence interval obtained from a bootstrap. Triangles mark the intercept of a linear regression line; filled triangles indicate a group-level effect different from zero (as in Supplementary Figure 6d). *** $p < 0.001$, ** $p < 0.01$, * $p < 0.05$, n.s. $p > 0.05$.

Figure 5f shows that RT reduced observers' intrinsic serial biases while pupil responses generally promoted choice alternation. These results further dissociate these modulatory effects, in showing that they were "gated" by trial outcome in distinct ways: Large pupil-linked arousal pushed observers to increase their intrinsic serial bias after correct trials, as indicated by the positive correlation in **a**. After error trials, on the other hand, a correlation of the opposite sign was observed – indicating that across trial outcomes, these two effects nullified and lead to an overall boost in alternation. This stood in sharp contrast to the group-level effect of RT, which predicted a reduction in intrinsic serial bias across the group. This effect was strongly present after error trials (**b**), suggesting an adaptive control mechanism could be at work only after negative feedback is received. After correct trials, high RTs indicated a slight reduction in bias, but this negative correlation was not significant across the group.
